# Liver δ-Aminolevulinate Dehydratase Activity is Inhibited by Neonicotinoids and Restored by Antioxidant Agents

**DOI:** 10.3390/ijerph111111676

**Published:** 2014-11-13

**Authors:** Elisa Sauer, Angela M. Moro, Natália Brucker, Sabrina Nascimento, Bruna Gauer, Rafael Fracasso, Adriana Gioda, Ruy Beck, José C. F. Moreira, Vera Lucia Eifler-Lima, Solange Cristina Garcia

**Affiliations:** 1 Laboratory of Toxicology (LATOX), Department of Clinical and Toxicology Analysis, Federal University of Rio Grande do Sul, Porto Alegre, RS 90610000, Brazil; E-Mails: elisa-sauer@hotmail.com (E.S.); angelammoro@yahoo.com.br (A.M.M.); nataliafarma@hotmail.com (N.B.); sabrinascimento@hotmail.com (S.N.); bruna_gauer@hotmail.com (B.G.); rafael.fra@hotmail.com (R.F.); 2 Institute of Cardiology, University Foundation of Cardiology, Porto Alegre, RS 90040371, Brazil; 3 Laboratory of Toxicology, Center of Biological and Health Sciences University of Caxias do Sul, Caxias do Sul, RS, 96815900, Brazil; 4 Post-Graduate Program Pharmaceutical Sciences, Federal University of Rio Grande do Sul, Porto Alegre, RS 90610000, Brazil; 5 Department of Clinical and Toxicology Analysis, Federal University of Santa Maria, RS 97105900, Brazil; 6 Department of Chemistry, Pontiﬁcal Catholic University of Rio de Janeiro (PUC-Rio), Rio de Janeiro, RJ 22453900, Brazil; E-Mail: agioda@hotmail.com; 7 Department of Production and Drug Control, Federal University of Rio Grande do Sul, Porto Alegre, RS 90610000, Brazil; E-Mail: ruy.beck@ufrgs.br; 8 Department of Biochemistry, Federal University of Rio Grande do Sul, Porto Alegre, RS 90035003, Brazil; E-Mail: 00006866@ufrgs.br; 9 Department of Production of Source-Material, Federal University of Rio Grande do Sul, Porto Alegre, RS 90610000, Brazil; E-Mail: veraeifler@ufrgs.br

**Keywords:** neonicotinoids, δ-ALA-D activity, toxicity, antioxidants

## Abstract

Neonicotinoids represent the most used class of insecticides worldwide, and their precursor, imidacloprid, is the most widely marketed. The aim of this study was to evaluate the effect of imidacloprid on the activity of hepatic δ-aminolevulinate dehydratase (δ-ALA-D), protective effect of potential antioxidants against this potential effect and presence of chemical elements in the constitution of this pesticide. We observed that δ-ALA-D activity was significantly inhibited by imidacloprid at all concentrations tested in a dose-dependent manner. The IC_50_ value was obtained and used to evaluate the restoration of the enzymatic activity. δ-ALA-D inhibition was completely restored by addition of dithiotreitol (DTT) and partly by ZnCl_2,_ demonstrating that the inhibition occurs by oxidation of thiol groups and by displacement of the Zn (II), which can be explained by the presence of chemical elements found in the constitution of pesticides. Reduced glutathione (GSH) had the best antioxidant effect against to δ-ALA-D inhibition caused by imidacloprid, followed by curcumin and resveratrol. It is well known that inhibition of the enzyme δ-ALA-D may result in accumulation of its neurotoxic substrate (δ-ALA), in this line, our results suggest that further studies are needed to investigate the possible neurotoxicity induced by neonicotinoids and the involvement of antioxidants in cases of poisoning by neonicotinoids.

## 1. Introduction

The widespread use of pesticides in agriculture has resulted in an increase of environmental pollution and health risks for non-target organisms, culminating in cases of acute and chronic poisoning [1]. Neonicoitinoids are currently the most important chemical class of insecticides marketed worldwide since the synthesis of the pyrethroids. The neonicotinoids have similar structures to nicotine and act on its site of action at the nicotinic acetylcholine receptor (nAChR) [2]. These compounds are classified as N-nitroguanidines (imidacloprid, thiamethoxam, dinotefuran, and clothianidin) and N-cyanoaminides (acetamiprid and thiacloprid) [3]. In 1991, this new class of pesticides was introduced into the market in the form of its precursor imidacloprid (IMI, 1-(6-chloro-3-pyridylmethyl)-N-nitroimidazolidin-2-ylideneamine) [4]. Since then, the widespread development of additional neonicotinoids for protection of modern crops reflects the importance of this chemical class, which is used for crop protection against piercing–sucking insects of cereals, vegetables, tea and cotton, and for flea control in cats and dogs [5]. Neonicotinoid pesticides represent 17% of all processed insecticides on the global market, with the class precursor imidacloprid being the most commercialized, representing 41.5% of sales [3,4].

Recent studies have shown that pesticides induce toxicity by several mechanisms. Some research has demonstrated that the biotransformation of pesticides generates reactive oxygen (ROS) and nitrogen (NOS) species, and that these free radicals are associated with the toxicity induced by these pesticides culminating in development of oxidative stress [2]. Oxidative stress is characterized by an imbalance between the production of free radicals and antioxidant defenses of the organism. Increased production of ROS may result from pathological conditions and by action of xenobiotics such as pesticides inducing tissue damage in several organs such as the heart, brain, kidney and liver [2,6,7].

Taking into account that δ-aminolevulinate dehydratase (δ-ALA-D) or porphobilinogen synthase is a metalloenzyme with thiol groups (-SH) that requires zinc ions for its activity [8], this enzyme can be inhibited by substances that compete with zinc, or by substances that oxidize-SH groups. Therefore, the δ-ALA-D activity can be inhibited by oxidation by different soft electrophiles or chemical elements that compete with Zn(II) in its active site. Recent studies have shown that this enzyme is a marker protein of oxidative stress situations. Indeed, numerous researches have shown a negative correlation between enzyme activity and the occurrence of oxidative stress [9].

In vertebrates, the liver, with its high metabolic rates and high concentrations of enzymes from the endogenous antioxidant system is the main organ involved in detoxification of xenobiotics [10]. The aim of this study was to evaluate how the presence of imidacloprid affects the activity of hepatic δ-ALA-D *in vitro*. Furthermore, we evaluated the protective effect of antioxidant agents such as resveratrol, curcumin, ascorbic acid and GSH against the potential toxic effects caused by this pesticide. Additionally, the presence of chemical elements which could interfere with the enzymatic activity of δ-ALA-D or increase its toxicityand not declared in the composition of imidacloprid was determined.

## 2. Materials and Methods

### 2.1. Chemicals

Imidacloprid (IMI, 1-(6-chloro-3-pyridin-3-methyl)-N-nitroimidazolidin-2-ylidenamine, Evidence^®^, 70% technical grade was obtained from Bayer CropScience (Morrisville, NC, USA). δ-Aminolevulinic acid (δ-ALA), dithiotreitol (DTT), ascorbic acid (L-3-ketothreohexuronic acid lactone), L-glutatione reduced (GSH), resveratrol, curcumin, Bradford reagent and bovine serum albumin were purchased from Sigma (St. Louis, MO, USA). Zinc chloride (ZnCl_2_) was purchased from Proquimios (Bangu, RJ, Brazil). All chemicals and solvents used were of analytical reagent grade quality and were used as received. Twice-deionized water was used.

### 2.2. Animals

The study was conducted using male adult Wistar rats weighing 270 ± 60 g, aged 6–8 weeks obtained from the Fundação Estadual de Produção e Pesquisa em Saúde (FEPPS, Porto Alegre, Brazil), maintained at 22 ± 28 ºC under a 12/12 h light/dark cycle, receiving standard food and water *ad libitum*. The animals treatment was conducted in accordance with the ‘‘Guiding Principles in the Care and uses of Animals’’ [11]. This study was approved by the local Ethics Committee of Universidade Federal do Rio Grande do Sul, No. 18427.

### 2.3. Tissue Preparation

Rats (*n* = 4) were sacrificed with an overdose of ketamine and xylazine anesthesia. Each liver sample was divided into equal parts of 1 g and stored at −80 °C until homogenization. Liver samples were homogenized with 50 mM Tris-Cl, pH 7.4 (1/10, *w/v*) and kept on ice. Homogenates were centrifuged at 581 g for 10 min to yield a low-speed supernatant (S1) fraction. Freshly prepared S1 was used for δ-ALA-D assays to obtain IC_50_ values and for enzyme activity reversibility tests.

### 2.4. Protein

Protein levels from supernatants were determined according to Bradford [12], using bovine serum albumin as standard.

### 2.5. δ-ALA-D Activity

δ-ALA-D activity was assayed by the method of Sassa (1981) [13], with some modifications. After a pre-incubation period, enzymatic reaction was initiated by adding the substrate (δ-ALA) in the medium and incubating for 1 h at 37 °C. The incubation was stopped by adding trichloroacetic acid solution (10% TCA) with 10 mM HgCl_2_. Porphobilinogen (PBG) was mixed with modified Ehrlich’s reagent, and the color developed was measured photometrically (555 nm) against a blank. Results were reported as nmol PBG/mg protein/h. All experiments were performed four times, and 1 g of liver from each rat as used.

### 2.6. Inhibitory Effect of Imidacloprid to δ-ALA-D Activity and IC_50_ Determination

The effect of imidacloprid on the liver δ-ALA-D activity was determined in the presence of different concentrations of pesticide (2–40 mM). The freshly prepared S1 was pre-incubated at 37 °C for 10 min with imidacloprid and after this time, the substrate (δ-ALA) was mixed to start the reaction. After data evaluation the IC_50_ value was determined and utilized to study the protective effect of resveratrol, curcumin, ascorbic acid and GSH.

### 2.7. Effect of Dithiothreitol (DTT) and Zinc Chloride (ZnCl_2_)

We verified the effect of DTT (3 mM) and zinc chloride (ZnCl_2_) (100 mM) in restore δ-ALA-D inhibition caused by imidacloprid (IC_50_). For that, imidacloprid (at IC_50_ concentration) was pre-incubated with freshly prepared S1 of liver tissue containing DTT or ZnCl_2_ for 10 min at 37 °C. After this time the reaction was started by the addition of substrate (δ-ALA).

### 2.8. Effect of Resveratrol, Curcumin, Ascorbic Acid and Reduced Glutathione on Liver δ-ALA-D Activity in the Presence of Imidacloprid

The protective effects of curcumin, resveratrol, ascorbic acid and GSH were studied in the presence of imidacloprid (IC_50_ concentration). The freshly prepared (S1) hepatic tissue was pre-incubated at 37 °C for 10 min with imidacloprid plus resveratrol or curcumin (0.001, 0.1, 1, 5, 10, 100 and 1000 μM), and imidacloprid plus ascorbic acid or GSH (10, 100 and 1000 μM).

### 2.9. Quantification of Chemical Elements in Imidacloprid

The chemical elements ^75^As, ^27^Al, ^114^Cd, ^59^Co, ^53^Cr, ^65^Cu, ^208^Pb, ^60^Ni, ^55^Mn, ^202^Hg and ^88^Sr were quantified in samples of commercial imidacloprid by inductively coupled plasma mass spectrometry (ICP-MS: NexION 300X, PerkinElmer, Norwalk, CT, USA). For the measurement of chemical elements, 1.0 mL of 65% nitric acid PA (redistilled) was added to 100 mg of pesticide in a sterile polypropylene tube. The mixture was digested by heating at 95 °C for 8 h. The extracts were cooled at room temperature and the volume was made up to 10.0 mL with ultrapure water. Calibration was performed using standard solutions of 10 µg∙L^−1^ (Perkin Elmer 29 and Merck Titrisol) and acidified with bidistilled nitric acid. Calibration curve concentrations ranged from 5 µg∙L^−1^ to 80 µg∙L^−1^, and the internal standard used was rhodium at a concentration of 400 µg∙L^−1^ for calibration. Precision and accuracy of the analytical method were monitored through the use of reference standards that were analyzed in intervals of 15 samples. For differences greater than 10%, a new calibration was applied. The limit of detection (LOD) was calculated using the formula LOD = 3 × (SD/S) and the limit of quantification (LOQ) was determined by the formula LOQ = 10 × (SD/S), where SD represents the standard deviations of the readings of 10 blanks and S is the sensitivity of the analytical curve (slope).

### 2.10. Statistical Analysis

Data were expressed as mean ± S.D. Statistical analysis was performed by one-way ANOVA followed by Bonferroni’s post-hoc tests. Values of *p* < 0.05 were considered statistically significant. The IC_50_ value was determined by linear regression from individual experiments using GraphPad software (GraphPad software, San Diego, CA, USA).

## 3. Results

### 3.1. Inhibitory Effect of Imidacloprid on δ-ALA-D Activity and Determination of IC_50_

**Figure 1:**
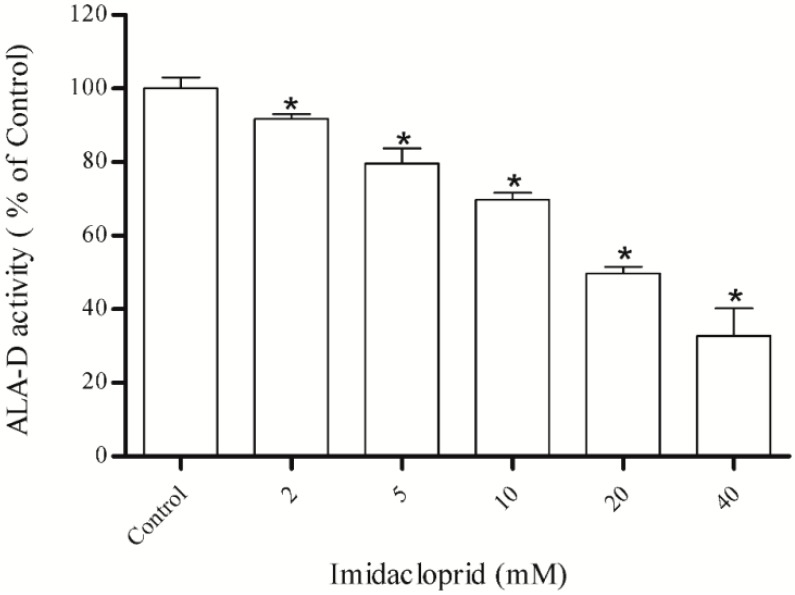
Effect of imidacloprid on δ-ALA-D activity from rat liver. Data are expressed as mean ± SD, (*n* = 4). δ-ALA-D activity of control (100%) was of 13.57 ± 0.17 (mean ± SD) nmol of porphobilinogen per mg protein per hour.

Hepatic δ-ALA-D activity was significantly inhibited by imidacloprid at the concentrations 2, 5, 10, 20 and 40 mM (Figure 1), in a dose-dependent manner. The IC_50_ value was 20.06 ± 0.17 mM. Next, the potential protective effect of the antioxidants curcumin, resveratrol, acid ascorbic and GSH was tested utilizing the IC_50_ value of imidacloprid.

* Denotes *p* < 0.05 as compared with the control, considering 100% (One-way ANOVA/ Bonferroni).

### 3.2. Effect of Dithiothreitol (DTT) and Zinc Chloride (ZnCl_2_)

**Figure 2:**
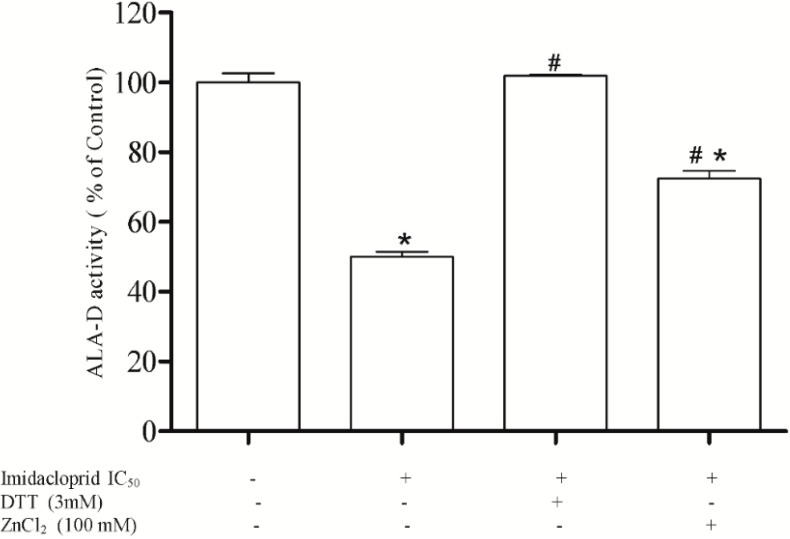
Effect of DTT and ZnCl_2_ as restoring agents δ-ALA-D inhibition caused by imidacloprid (IC_50_ = 20 mM). Data are expressed as mean ± SD, (*n* = 4).

The inhibitory effect of imidacloprid (20 mM) on hepatic δ-ALA-D activity was completely (100%) restored by the addition of DTT (3 mM) and partially restored (75%) by ZnCl_2_ (100 mM) when compared to the initial enzyme activity (Figure 2).

* Denotes *p* < 0.05 as compared with the control (100%) (One-way ANOVA/Bonferroni). # Denotes *p* < 0.05 as compared with the imidacloprid (20 mM) (One-way ANOVA/Bonferroni). The positive and negative signs mean added or not to the assay.

### 3.3. Effect of Resveratrol, Curcumin, Ascorbic Acid and Reduced Glutathione on Liver δ-AlA-D Activity in the Presence of Imidacloprid

**Figure 3:**
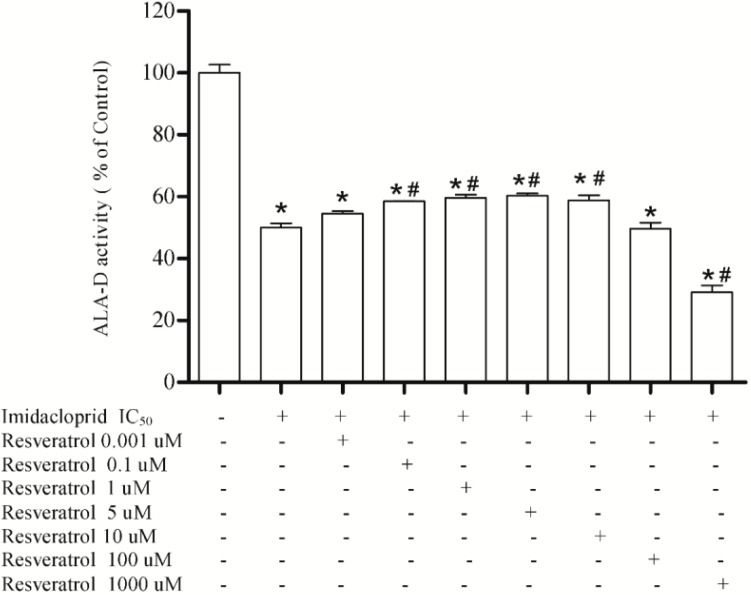
Effect of resveratrol (0.001, 0.1, 1, 5, 10, 100 and 1000 µM) as restoring agent for δ-ALA-D inhibition caused by imidacloprid (IC_50_ = 20 mM).Data are expressed as mean ± SD, (*n* = 4).

Results showed that resveratrol at 0.1, 1, 5, and 10 μM restored the δ-ALA-D activity inhibited by imidacloprid (20 mM) at 59%, 61%, 61% and 58%, respectively, when compared to the initial enzymatic activity. However, resveratrol at 100 μM did not restore the inhibitory effect of imidacloprid (20 mM) on δ-ALA-D activity, and at 1000 μM had an inhibitory effect on enzymatic activity, which decreased to 29% when compared to the baseline activity (Figure 3).

**Figure 4:**
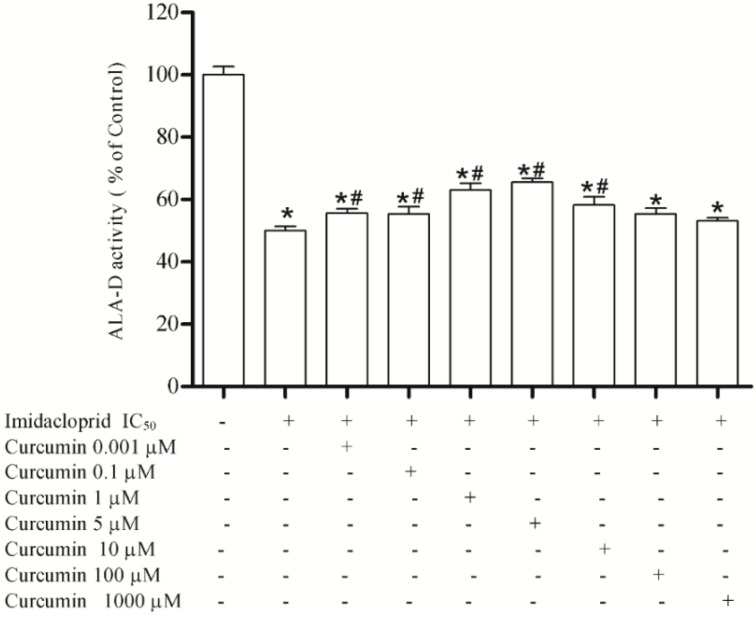
Effect of curcumin (0.001, 0.1, 1, 5, 10, 100 and 1000 µM) as restoring agent for δ-ALA-D inhibition caused by imidacloprid (IC_50_ = 20 mM). Data are expressed as mean ± SD, (*n* = 4).

Curcumin at 0.001, 0.1, 1, 5 and 10 μM restored the enzyme activity inhibited by imidacloprid (20 mM) at 55%, 55%, 63%, 65% and 58%, respectively, when compared to the initial enzyme activity, while at 100 and 1000 μM was not able to restore enzyme activity (Figure 4).

**Figure 5:**
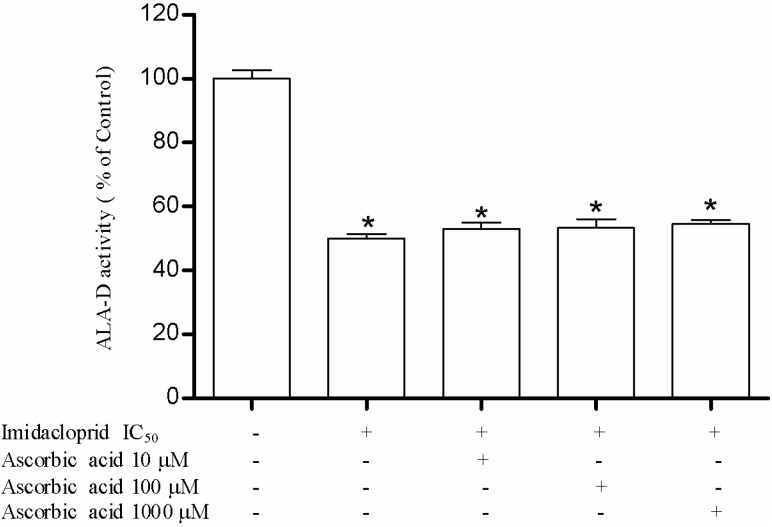
Effect of ascorbic acid (10, 100 and 1000 µM) as restoring agent for δ-ALA-D inhibition caused by imidacloprid (IC_50_ = 20 mM). Data are expressed as mean ± SD, (*n* = 4).

**Figure 6:**
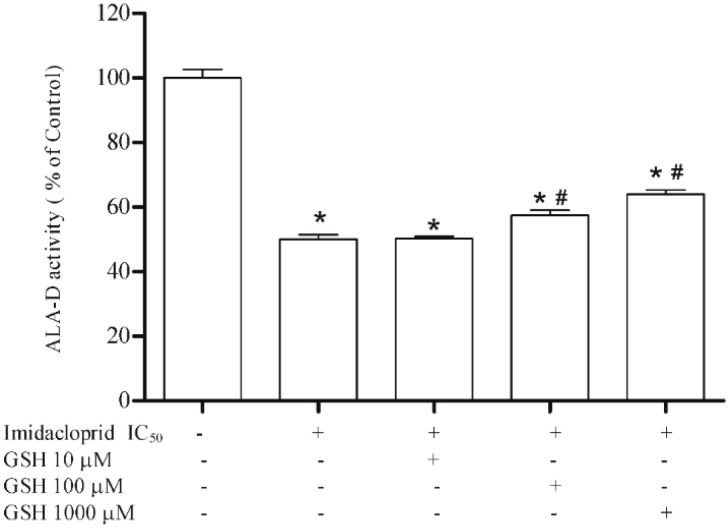
Effect of reduced glutathione (10, 100 and 1000 µM) as restoring agent for δ-ALA-D inhibition caused by imidacloprid (IC_50_ = 20 mM). Data are expressed as mean ± SD, (*n* = 4).

We verified that ascorbic acid treatment at 10, 100 and 1000 μM was not able to restore the enzymatic inhibition (Figure 5), and that reduced glutathione at 100 and 1000 μM partially restored the inhibition to 53% and 67% (Figure 6).

### 3.4. Determination of Chemical Elements in Imidacloprid

The concentrations of chemical elements analyzed in commercial imidacloprid are shown in Table 1.

* Denotes *p* < 0.05 as compared with the control (100%) (One-way ANOVA/Bonferroni). # Denotes *p* < 0.05 as compared with the imidacloprid (20 mM) (One-way ANOVA/Bonferroni). The positive and negative signs mean added or not to the assay.

* Denotes *p* < 0.05 as compared with the control (100%) (One-way ANOVA/Bonferroni). # Denotes *p* < 0.05 as compared with the imidacloprid (20 mM) (One-way ANOVA/Bonferroni). The positive and negative signs mean added or not to the assay.

* Denotes *p* < 0.05 as compared with the control (100%) (One-way ANOVA/Bonferroni). The positive and negative signs mean added or not to the assay.

* Denotes *p* < 0.05 as compared with the control (100%) (One-way ANOVA/Bonferroni). # Denotes *p* < 0.05 as compared with the imidacloprid (20 mM). The positive and negative signs mean added or not to the assay.

**Table 1:** Concentration of chemical elements present in imidacloprid quantified by ICP-MS.

Chemical elements	Concentration (mg∙L^−1^)
Aluminum	8.43
Arsenic	0.28
Cadmium	0.02
Cobalt	0.05
Copper	2.98
Chromium	2.81
Lead	0.11
Manganese	77.62
Mercury	0.11
Nickel	0.51
Strontium	3.27

## 4. Discussion and Conclusions

Currently, neonicotinoids are classified by the Environmental Protection Agency (EPA) system as toxicity class II and/or class III agents [14], because they block a specific neuron pathway that is more abundant in insects than warm blooded animals, so the toxicity of these insecticides is more selective to insects than mammals [2]. However, these insecticides affect insects by interfering with nAChRs, suggesting that these receptors may also be a target in mammals. Moreover, there are reports of neonicotinoid intoxications, with clinical manifestations including nausea, vomiting, drowsiness, disorientation, dizziness, oral and gastroesophageal erosions, hemorrhagic gastritis, productive cough, fever, leukocytosis, muscle weakness, hypothermia and convulsions [15,16,17]. Calderón-Segura *et al.* [3] demonstrated that commercial neonicotinoid formulations induced DNA damage, reduced the viability of human lymphocytes and caused cell death. Birsen [18] demonstrated that treatment with the neonicotinoid thiacloprid results in increased oxide nitric (NO) levels in rat polymorphonuclear leukocytes and plasma. El-Gendy *et al.* [2] showed that imidacloprid treatment of rats induced a marked increase in the hepatic lipid peroxidation. Moreover another study found changes in liver enzymes, oxidative stress markers, induction of pro-inflammatory cytokines and NO levels in the brain and liver of imidacloprid-exposed rats [5].

In the present study, we demonstrated for the first time that the neonicotinoid imidacloprid is able to inhibit the activity of δ-ALA-D in liver tissue. This enzyme has thiol groups (-SH) in its active site, which are essentially involved in the coordination of Zn(II) ions, and the proximity between these groups renders the enzyme easily oxidizable [19,20]. The δ-ALA-D inhibition by toxic agents, such as imidacloprid, contributes to the increase of circulating levels of the substrate aminolevulinic acid (ALA). ALA is a pro-oxidant compound and a weak γ-aminobutyric acid (GABA) agonist, which is responsible for decreasing GABA release by presynaptic inhibition and may cause neurotoxicity [21]. Additionally, other experimental studies have shown that ALA possibly presents other central nervous system effects such as induction of free radical formation, effects on the uptake and release of glutamate, inhibition of Na^+^, K^+^, -ATPase activity and seizures induced by glutamatergic mechanisms [22]. Patients affected by disorders characterized by increased levels of aminolevulinic acid, such as occurs in porphyrias, present acute attacks characterized by neurological manifestations, including seizures and psychiatric manifestations such as hysteria, anxiety and depression [22].

Furthermore, δ-ALA-D is directly involved in the synthesis of grouping tetrapyrroles, such as heme. The enzymatic inhibition caused by the addition of imidacloprid may interrupt or interfere with the synthesis of heme groups resulting in damage to the cell metabolism and injury to the health of non-target organisms exposed to pesticides. These alterations may occur both by induction of neurotoxicity or producing an increase in oxidative effects due to the pro-oxidant activity of the accumulated enzyme substrate ALA [9].

Recent studies have shown that the enzymatic activity of δ-ALA-D can be used as a biomarker of pro-oxidant situations because it is extremely susceptible to oxidizing agents and must be in the reduced state to catalyze the substrate formation [9]. Several studies have shown a negative correlation between the activity of δ-ALA-D and the occurrence of oxidative stress. Previous studies have shown that δ-ALA-D activity was inhibited in hemodialysis patients [23,24], in patients after bone marrow transplantation [25] and in patients with diabetes [26]. Furthermore, the activity of δ-ALA-D was inhibited after exposure to other pro-oxidant situations such as hyperoxygenation [27] and after exposure to paints, which contain a broad mixture of solvents [28]. Therefore, based on previous studies that demonstrate that the enzyme is a good marker for pro-oxidant conditions and oxidative stress, we can infer that imidacloprid is a compound capable of causing oxidative stress since it was able to significantly inhibit the enzymatic activity of δ-ALA-D.

Since δ-ALA-D activity may be inhibited by compounds that oxidize the –SH groups or which remove Zn(II) from the enzyme structure, we studied the possible mechanism(s) of imidacloprid toxicity. In this line, it was observed that the enzymatic inhibition caused by imidacloprid was restored by the addition of dithiothreitol (DTT), which is a dithiol that possesses the ability of protecting δ- ALA-D against inhibition by sulfhydryl oxidizing agents [29]. On the other hand, we verified that ZnCl_2_ was partially able to restore δ-ALA-D activity, thus we propose that the mechanism involved in the inhibitory effect of imidacloprid on δ-ALA-D activity is also by zinc displacement of the enzyme structure. Chemical elements found in the composition of the pesticide may be contributing to the direct oxidation of thiol groups ‒SH or interacting by facilitating the displacement of Zn(II). Thus, the toxic effects caused by pesticide on the enzyme activity are not only resulting from the active principle imidacloprid, but also of chemical elements presents in the formulation, which are not declared on the product label. A study has shown strong reactivity *in vitro* of some chemical elements such as Pb, Hg, Cd, As and Al, which are chemical elements found in the imidacloprid formulation, with–SH, causing inhibition of enzyme activity [9]. Besides that, high concentrations of Mn were found in the imidacloprid formulation. Mn is an essential metal necessary for normal functioning of many biological processes such as energy metabolism and the immune system [30]. Additionally, Mn can be found in the brain as an important co-factor of enzymes involved in the antioxidant system [31]. However, despite its essentiality, several psychomotor and psychiatric disorders are associated with exposure to high levels of Mn, such as Parkinson’s disease symptoms, cognitive deficits, memory loss, impaired learning demonstrating its neurotoxic potential [32]. Therefore, the presence of this metal in high concentrations in the imidacloprid formulations could potentiate the neurotoxic effects resulting from the inhibition of the enzymatic activity of ALA-D caused by this pesticide.

We used resveratrol and curcumin as potential antioxidants to reverse the inhibition of δ-ALA-D caused by imidacloprid and other chemical agents found in the pesticide. Resveratol (3,5,4'-tri- hydroxystilbene) is a phytoalexin found in grapes and in foods such as peanuts, blueberries, and red wines, and along with curcumin is a polyphenolic compound. Curcumin (diferuoylmethane) is the most active component of turmeric, an agent derived from dried rhizomes of the plant turmeric (*Curcuma longa* L.), and is one of the most recently studied chemopreventive compounds [33,34,35]. As shown in the results, both curcumin and resveratrol at low concentrations were effective at partially restoring enzyme activity, in other words, they are able to restore enzyme activity due to their antioxidant activity, protecting the enzyme from the oxidative effects of the pesticide. On the other hand, the highest concentrations of resveratrol tested (100 and 1000 μM) were unable to restore enzyme activity. Indeed Stooco *et al.* [36] have shown that at high concentrations these antioxidants may present pro–oxidant effects. In addition, the concentration 1000 μM caused an increase in enzymatic inhibition induced by the pesticide, demonstrating a pro-oxidant effect of resveratrol, which was not observed with curcumin. Our findings corroborate some reports in the literature, where both were recognized as potential antioxidants at low concentrations, because they present the ability to eliminate free radicals and increase antioxidant effects [33,34,35].

Moreover, we also used GSH and ascorbic acid to test their influence on enzyme activity inhibited by the pesticide. Ascorbic acid is widely known due to its antioxidant properties. It is an excellent source of electrons, acting as a donor of electrons and neutralizing free radicals. Due to its solubility in water, ascorbic acid promotes an antioxidant effect both inside and outside the cell and prevents free radical damage [37,38]. Ascorbic acid may scavenge peroxyl radicals and inhibit cytotoxicity induced by oxidants [39]. El-Gendy *et al.* [2] demonstrated that ascorbic acid promotes protective effects against toxicity induced by imidacloprid in rats as observed by some markers of oxidative stress such as MDA, GSH, CAT, SOD and GPx. In our study, ascorbic acid was not able to restore enzyme activity inhibited by the pesticide. However El-Gendy *et al.* [2] also showed that pre-treatment with ascorbic acid is most effective when compared to post-treatment. In our study, both pesticide and ascorbic acid were incubated simultaneously, and probably would a pre-incubation with ascorbic acid is necessary to obtain the protective effects of this antioxidant.

GSH is a non-protein thiol widely distributed in animal tissues, and closely linked to the antioxidant cell response against the toxic effects of reactive oxygen species [40]. In the present study, GSH demonstrated its antioxidant effect when it was able to partially reverse the enzymatic activity inhibited by imidacloprid at the two highest concentrations tested (100 and 1000 μM). Other studies have shown the benefits of GSH treatment, resulting in a reduction in the oxidant burden at the alveolar epithelial surface in the idiopathic pulmonary fibrosis [41], attenuation of reactive oxygen species generation and increased antioxidant defenses [42], attenuation of lipid peroxidation [43] and prevention of neurotoxicity induced by treatment with oxaliplatin without reducing the clinical activity of oxaliplatin [44].

Our study has some limitations. The imidacloprid concentration range used in our study is very high and may not represent environmental relevance. On the other hand, we aimed to investigate the potential toxic effects of this pesticide in order to assess the risks in cases acute occupational exposure, as well as in cases of accidental or intentional poisoning. A study by Kimura-Kuroda *et al.* [45], evaluated the effect of imidacloprid in cerebellar neurons and found that in concentrations more than 1 M the pesticide exerts excitatory effects of nicotinic acetylcholine receptors (nAChRs) and thus may harm human health, especially in the developing brain. The concentration used in this study was much lower than those used in our study, however, Kimura-Kuroda *et al.* used a primary cell culture model that mimics the conditions of the body.

In view of our data, this study supports the evidence that imidacloprid, an insecticide widely used and classified as being of low toxicity, induces oxidative damage since it significantly inhibited the enzymatic activity of δ-ALA-D. Moreover, the enzymatic inhibition of δ-ALA-D could contribute to accumulation of its enzymatic substrate δ-ALA that may rapidly oxidize to generate ROS such as superoxide ions, hydroxyl radicals and hydroxyl peroxides generating a vicious pro-oxidant cycle [9] that could promote neurotoxic effects [21]. The inhibition was reversed completely after treatment with the reducing agent DTT and the partial restoration by the use of ZnCl_2_ indicates that the enzyme inhibition does not occur only by oxidation of thiol groups, but also by Zn(II) displacement, which is possibly related to the presence of various chemical elements in the pesticide formulations. Therefore, the toxicity demonstrated by the studied pesticide could be associated to the active principle, imidacloprid, but also to the presence of different chemical elements that are not declared in its formulation, which can contribute to potentiate the toxicity caused by imidacloprid. Knowing that some of the chemical elements found in the pesticide formulation have an influence on the enzymatic activity of δ-ALA-D, future studies using these chemicals elements, mainly manganese, found in pesticide formulations are important to evaluate the effect of these elements on the inhibition enzymatic activity of δ-ALA-D, a point that can be a limitation in our study. Considering our results together with previous findings in the literature, it could be stated that this neonicotinoid, despite its low toxicity classification, should be investigated carefully because it may cause serious damages to non-target organisms, especially after chronic exposure. Hepatio-protective effects were observed in imidacloprid-induced liver toxicity after the use GSH at 1000 μM followed by curcumin at 5 μM, which appeared as the second most powerful antioxidant and resveratrol at 5 and 10 μM concentrations. Therefore, it could be suggested that these compounds could contribute to prevention of inhibition caused by this pesticide along with other chemicals present in the formulation on δ-ALA-D activity.
